# Cork Oak Vulnerability to Fire: The Role of Bark Harvesting, Tree Characteristics and Abiotic Factors

**DOI:** 10.1371/journal.pone.0039810

**Published:** 2012-06-28

**Authors:** Filipe X. Catry, Francisco Moreira, Juli G. Pausas, Paulo M. Fernandes, Francisco Rego, Enrique Cardillo, Thomas Curt

**Affiliations:** 1 School of Agriculture, Centre for Applied Ecology, Technical University of Lisbon (CEABN-ISA-UTL), Lisbon, Portugal; 2 Desertification Research Centre, Spanish National Research Council (CIDE-CSIC), Montcada, Valencia, Spain; 3 Centre for the Research and Technology of Agro-Environmental and Biological Sciences, University of Trás-os-Montes e Alto Douro (CIFAP-UTAD), Vila Real, Portugal; 4 Institute for the Wood, Cork and Charcoal (IPROCOR), Badajoz, Spain; 5 Mediterranean Ecosystems and Risks Research Unit, National Research Institute of Science and Technology for Environment and Agriculture (EMAX-Irstea), Aix-en-Provence, France; Lakehead University, Canada

## Abstract

Forest ecosystems where periodical tree bark harvesting is a major economic activity may be particularly vulnerable to disturbances such as fire, since debarking usually reduces tree vigour and protection against external agents. In this paper we asked how cork oak Quercus suber trees respond after wildfires and, in particular, how bark harvesting affects post-fire tree survival and resprouting. We gathered data from 22 wildfires (4585 trees) that occurred in three southern European countries (Portugal, Spain and France), covering a wide range of conditions characteristic of Q. suber ecosystems. Post-fire tree responses (tree mortality, stem mortality and crown resprouting) were examined in relation to management and ecological factors using generalized linear mixed-effects models. Results showed that bark thickness and bark harvesting are major factors affecting resistance of Q. suber to fire. Fire vulnerability was higher for trees with thin bark (young or recently debarked individuals) and decreased with increasing bark thickness until cork was 3–4 cm thick. This bark thickness corresponds to the moment when exploited trees are debarked again, meaning that exploited trees are vulnerable to fire during a longer period. Exploited trees were also more likely to be top-killed than unexploited trees, even for the same bark thickness. Additionally, vulnerability to fire increased with burn severity and with tree diameter, and was higher in trees burned in early summer or located in drier south-facing aspects. We provided tree response models useful to help estimating the impact of fire and to support management decisions. The results suggested that an appropriate management of surface fuels and changes in the bark harvesting regime (e.g. debarking coexisting trees in different years or increasing the harvesting cycle) would decrease vulnerability to fire and contribute to the conservation of cork oak ecosystems.

## Introduction

Many forest and woodland ecosystems in the world provide a range of social, economic and ecological services, far beyond timber exploitation. Bark is one of the most important non-timber forest products in many countries worldwide and it is periodically harvested from many tree species [1]–[5]. Tree bark provides protection against desiccation, fire, insects and diseases, and plays a key role in the transportation of nutrients from leaves to roots through the phloem tissues, thus bark harvesting may induce internal tree stress and increase vulnerability to external agents [6]–[10]. In fact, bark extraction has been reported to alter tree survival, growth and reproduction in a range of species worldwide [4]–[7], [10]. However, little is known about the impacts of bark harvesting on tree vulnerability to fire, which is especially relevant in the light of current changes in climate and fire regimes.

**Figure 1:**
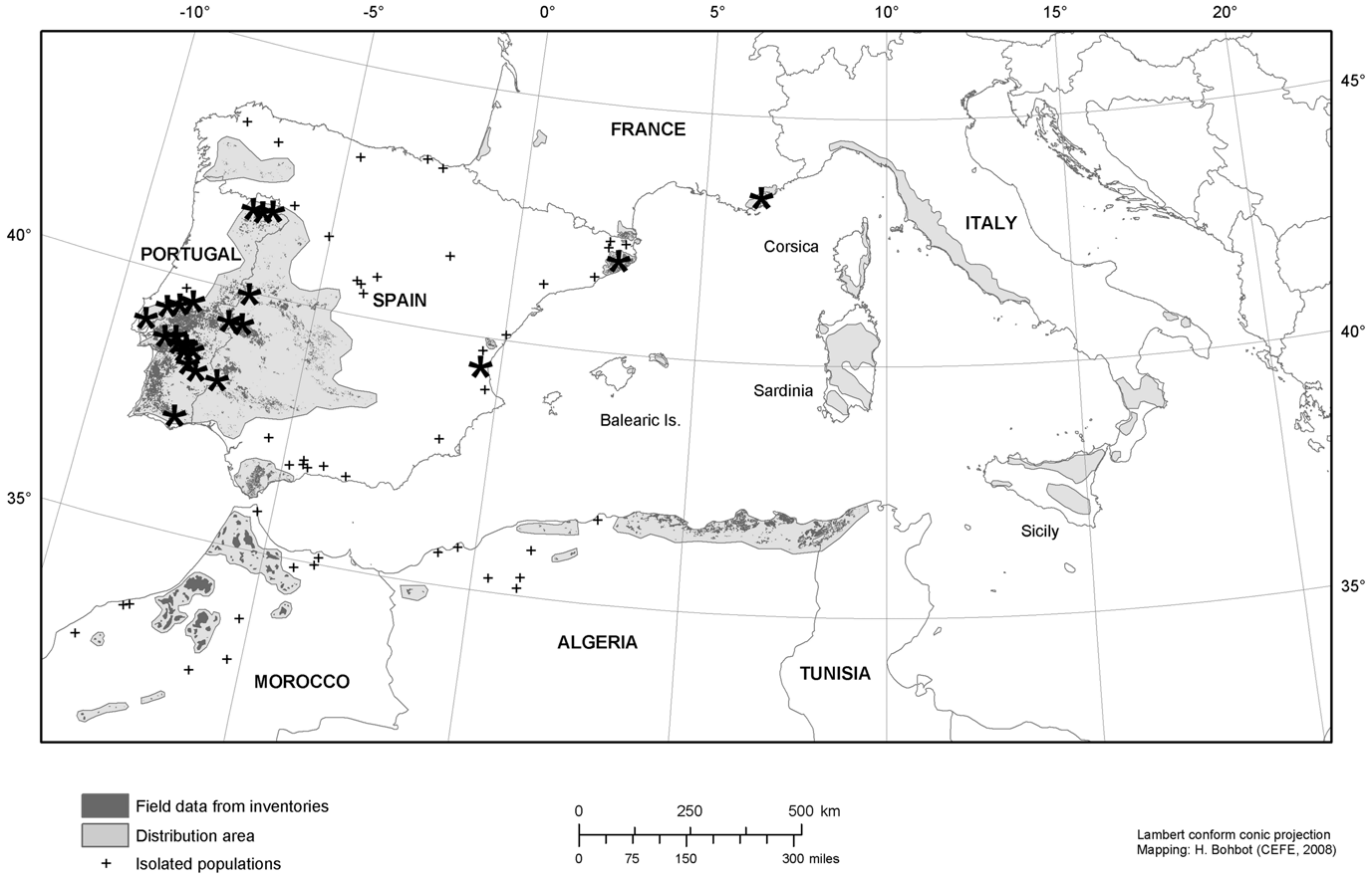
Distribution of *Q. suber* and location of the 22 study sites. General distribution of *Q. suber* in the Mediterranean Basin (in grey; reproduced from Pausas, Pereira & Aronson 2009 with permission from Island Press, Washington, DC) and location of the 22 study sites (black stars).

A prominent case of a tree whose bark is recurrently harvested is cork oak, *Quercus suber* L. Cork oak ecosystems span from open savannas to closed forests and cover nearly 2.5 million hectares in the western Mediterranean Basin [11] (Figure 1). The bark of *Quercus suber* (the cork) has excellent insulation properties and can grow up to 30 centimetres thick [12]. Possessing a thick bark is a clear mechanism for protecting the cambium from the heat generated by fires [13], [14] and has been evolutionarily linked to fire [15]. Indeed, *Q. suber* has been considered a highly fire-resilient species, being the only European tree with stem and crown resprouting capability (through epicormic buds) after intense crown-fires [16], [17]. Thick bark is a fire adaptation that has also appeared in other plants living in fire-prone ecosystems from other continents (convergent evolution, [14]).

Currently, cork is the second most important marketable non- timber forest product in the western Mediterranean [18], and the world cork market exports represents nearly US$2 billion annually [19]. *Q. suber* is particularly important in the Iberian Peninsula, which holds 82% of the world's cork production [20]. Cork is a renewable natural resource constituting a valuable and versatile raw material for industry, and is used for a large variety of products, with wine bottle stoppers representing most of the cork market value. Because of its economic value, the cork of *Q. suber* is periodically harvested, starting when trunk diameter reaches about 20 cm and with subsequent harvests at 9–15 year intervals. In addition to the economic importance of cork, these forest areas often alternate with multi-purpose farmland systems, which integrate extensive agriculture, forestry, grazing, and other uses. The cork oak ecosystems are also recognized for their remarkable ecological value, providing habitat for several threatened species and being protected by international legislation [8], [20], [21].

Cork oak can withstand recurrent bark harvesting (a tree can be stripped 12–20 times during its life), but such activity causes some undesirable effects. Probably the most important of these effects is the reduced protection against external agents, particularly wildfires, increasing tree vulnerability. Additionally, cork harvesting has other consequences including water losses through the stripped stem, reduction of nutritional functions and a very high consumption of stored reserves [3], [12], which may increase tree stress and further increase vulnerability to fire.

Wildfires are an increasing concern in the Mediterranean Basin [22], with nearly half a million hectares burned every year [23]. For instance, in Portugal (the world leading country in *Q. suber* area and cork production), 15–20% of *Q. suber* forests have burned since 1990. Furthermore, fire risk is likely to increase in the future (along with drought and diseases) due to climate change [25]–[27]. In spite of being considered a highly fire-resilient species, local studies suggest that *Q. suber* responses to fire are variable [16], [28]–[32], but the reasons for such variability are poorly understood. Our hypothesis is that tree resistance and resilience depends on management activities, particularly those affecting the individual traits that confer protection against external agents and the tree physiological status, e.g. debarking. However, these factors may also vary in relation to individual tree characteristics, e.g. size [32], [33], fire behaviour [26]–[32] and environmental conditions (e.g. precipitation, season) that influence tree vigour and phenology [7], [9]. Specifically, the aims of this paper are:

1. To test whether management of cork oak trees, in particular bark harvesting, is a key factor influencing post-fire tree vegetative responses across a wide geographical area. We predict that tree vulnerability to fire will decrease with increasing bark thickness and will increase with bark harvesting, as harvesting not only reduces bark thickness, but also increases tree stress.
2. To explore the role of fire severity and tree size on post-fire *Q. suber* responses. Tree resistance to fire is expected to decrease with increasing fire severity and with tree size as resprouting ability tends to decrease with size in other oaks.
3. To explore to what extent abiotic factors such as topography, climate or fire season explain between-site variations in the ability of *Q. suber* to cope with fire. Higher tree susceptibility is expected under unfavourable environmental conditions, particularly those related to drought. In addition, early season fires, i.e. when trees are flowering or actively growing, may have a higher negative impact on resprouting than later fires.

To attain these objectives we gathered a large data set on post-fire *Q. suber* responses across a wide range of ecological conditions and management frameworks encompassing most of the species distribution. We present models that accurately describe the factors increasing post-fire vulnerability of *Q. suber*. Such models provide the scientific basis for improving the management and enhancing the conservation of cork oak ecosystems.

## Methods

### Ethics statement

All necessary permits were obtained for the field work, through contacts with land owners and local forest associations.

### Study areas

We compiled data on post-fire tree responses (i.e. survival and vegetative regeneration) from 22 wildfires that occurred between 1994 and 2006 in the western Mediterranean (Figure 1). Most study sites (16) were located in Portugal, the country where *Q. suber* is more abundant, and the remaining were located in Spain (5) and France (1). The sample covers a wide range of ecological and management conditions, from open woodlands (savanna-like systems) to dense forests (see Table 1 and Tables S1 & S2 in Supporting Information). All sites were under Mediterranean climate, with specific conditions ranging from inland regions with lower annual precipitation (550 mm) and higher temperature, to coastal regions with higher rainfall (1100 mm) and milder temperature. *Q. suber* was the dominant tree species in most sites and the understorey was composed of a shrub-herbaceous layer that favoured fire spread.

**Table 1:** Summary of the variables assessed.

Variable (Code)	Units	Level	Spatial scale (database)	Mean (Range)
Bark thickness (BT)	mm	Tree	Overall	21 (0–140)
Diameter at breast height (DBH)	cm	Tree	Overall	21 (0.5–133)
Exploitation status (Ex)	2 categories^2^	Tree	Overall	-
Tree height (TH)	m	Tree	Overall	7.1 (1.2–21)
Tree response type (R)	4 categories^3^	Tree	Overall	-
Percentage of char height (PCH)	%	Tree	West Iberia	88 (0–100)
Fire season (FS)	2 categories^4^	Site	Overall	-
Ecological region (ER)	3 categories^1^	Site	Overall	-
Mean annual precipitation (AP)	mm	Site	Overall	732 (550–1100)
Mean annual temperature (AT)	°C	Site	Overall	15.1 (11.5–18.0)
Mean elevation (E)	m	Site	Overall	359 (6–650)
Mean proportion of trees in unfavourable aspects (UA)	%	Site	Overall	50 (0–100)
Mean slope (S)	%	Site	Overall	18 (0–55)
Time since fire (TSF)	years	Site	Overall	2 (1–4)

1 Ecological region (RE) categories (EEA 2003): Iberian sclerophyllous and semi-deciduous forests (code 159), Northeastern Spain & Southern France Mediterranean (code 162), Southwest Iberian Mediterranean sclerophyllous and mixed forests (code 168); ^2^ Exploitation status (Ex) categories: exploited or unexploited; ^3^ Tree response type (R) categories: dead, resprouting from the base only, resprouting simultaneously from the base and the crown, or resprouting from the crown only; ^4^ Fire season (FS) categories: early summer season or late season.

### Data collection

In most sites (73%) we used a regular grid (500×500 m) of points covering the burned area and defined a circular sampling plot (50 m of radius, 7850 m2) around each point. In plots with 30 oaks or less, all trees inside the plot were assessed; otherwise, we laid out up to four 50-m perpendicular strip transects and sampled trees (starting with north and proceeding in a clockwise direction) until obtaining of the 30 trees per plot. In the remaining sites, plots were smaller (375 and 400 m^2^ in Spain and France, respectively), and all trees inside each plot were sampled. In total, 203 plots were sampled across the 22 study sites. Each site included up to 40 plots (average 9 plots per site; see Table S1). The database was organized at two different spatial levels, overall and West Iberia databases, and the variables sampled depended on that level (Table 1).

The overall database included all the 4585 *Q. suber* trees sampled. For each tree, bark thickness (BT) was estimated as the average of four measurements at breast height made with a bark gauge at opposite sides of the trunk. Tree bark exploitation status (Ex) was defined as a binary variable (exploited or unexploited) based on the presence of harvesting marks on the stem. For the exploited trees, time since harvesting was not directly addressed as such information was not available for all trees. However time since harvesting (i.e., bark age) is naturally related with bark thickness in exploited trees, as bark regrows after debarking. We confirmed this by analysing such relation in a sample of 491 exploited trees for which the harvesting year was known. Bark age ranged from 0, corresponding to trees debarked in the year of the fire, to 13 years. We found a significant correlation between BT and cork age at the time the fire occurred (r = 0.67, *P*<0.001).Tree size variables included total tree height (TH), and diameter at breast height inside bark (DBH). The post-fire response type of each tree (R) was recorded as dead, resprouting from the base (stump) only, resprouting simultaneously from the base and the crown, or resprouting from the crown only, following a decreasing gradient of fire-inflicted damage [32]. Dominant slope (percentage), elevation (meters) and aspect were measured in each plot. Aspect was simplified as a binary factor, i.e., unfavourable (S, SE or SW) or otherwise, because of the drier conditions in southern aspects (e.g. [34]). Then, for each site, mean slope (S), mean elevation (E), and the proportion of trees in unfavourable aspects (UE) were calculated. We also used geographic information systems (GIS) to locate the study sites and obtain additional data at the site-level, namely mean annual precipitation (AP), mean annual temperature (AT) and ecological region (ER) [35]. Three ecological regions were considered, i.e., forest with Atlantic-maritime influence (10 sites, all in Portugal), forest with continental influence (9 sites, from Portugal and Spain), and forest with Mediterranean-maritime influence (3 sites, from Spain and France) (see Table 1).

The wildfire date was obtained from official Forest Services fire databases, and classified into two fire seasons (FS): early (June and July) and late (August and September, including one winter fire). The time between the fire date and tree measurements (TSF) was also registered (see Tables 1 and S1).

The West Iberia database is a subset from the overall database and included data from the western Iberian Peninsula (3850 trees sampled in 19 sites). In this region, in addition to the previously mentioned variables, the maximum tree char height (the vertical extent of trunk blackening) was measured for each tree, and used as an indicator of fire severity and potential injury. The derived variable PCH (percentage of tree char height) expresses char height relative to total tree height.

### Data analysis

The main data analysis was performed at the two different spatial scales (overall and west Iberia) using binomial generalized linear mixed-effects models (GLMM) with a logit link [36], [37] and site as the random factor. As dependent variables we used the following tree responses (as binary variables; i.e., yes/no): i) individual mortality, i.e. mortality of all aboveground and belowground organs (tree death), ii) stem and tree mortality i.e. death of at least the aboveground biomass (top-kill hereafter), and iii) crown resprouting only (i.e., surviving trees and stems with epicormic resprouting). These post-fire responses were examined in relation to the different explanatory variables that were sampled at the tree-level (BT, Ex, DBH, and PCH for West Iberia); the interactions between BT and Ex, and DBH and Ex, were also examined to test the hypothesis that the tree responses to BT and DBH might be different according to exploitation status. All GLMM analyses were performed using the lme4 package from R [36], [38].

For each response type and spatial scale combination (total of 6 models) we started with a GLMM including all tree-level variables and used backward elimination to select the most important ones [37]. Model selection was performed by removing in each step the variable that explained less deviance, until all remaining variables in the model were significant (*P*<0.05). The final model was further evaluated by adding all significant variables sequentially and tested with a likelihood ratio test. Prior to GLMM, correlation between variables was checked using the Pearson correlation coefficient (between continuous variables) and the point biserial correlation (between continuous and dichotomous variables). The only highly correlated pair of variables was DBH and TH (r = 0.69), and since DBH was easier to assess in the field and more accurate, TH was excluded from the model building process.

Model performance was assessed by calculating the area under the receiver operating characteristics (ROC) curve [39], [40]. The ROC method has advantages in assessing model performance in a threshold-independent fashion, being independent of prevalence [41]. Usually area under curve (AUC) values of 0.5–0.7 are taken to indicate low accuracy, values of 0.7–0.9 indicate useful applications and values above 0.9 indicate high accuracy [42]. The Nagelkerke pseudo-R^2^ [43] was used as an indicator of the proportion of variance explained by the models.

In order to explore to what extent random (site-level) effects in the GLMM could be related to environmental variables, we extracted the coefficients for each site for the different models [36] and correlated them with the environmental variables in each site (Pearson correlation coefficient between continuous variables and point biserial correlation between continuous and dichotomous variables).

### Bark measurement constraints

One of the potential problems with bark thickness measurement after fire is the possibility of under- or overestimation. Specifically, underestimation could be caused by some reduction in BT because of external cell layers consumption during the fire. On the other hand, the BT of surviving stems could have increased in the period between the fire and the field measurements because of bark growth, leading to BT overestimations. However, both under- and overestimation, are unlikely to be important because cork is a very good insulation material, that does not burn easily, and because bark growth is expected to stop or strongly decrease during the first few years after the fire. Nevertheless, to evaluate this potential limitation we did a sensitivity analysis where BT (at the time of the fire) was estimated based on these expected losses and gains (see details in Text S1).

## Results

### General patterns of post-fire cork oak responses

From all 4585 *Q. suber* trees sampled, nearly 16% died after fire (individual mortality) and 13% lost their crowns showing basal resprouting only, totalling 29% of top-killed trees. The remaining trees regenerated their crown, with 56% resprouting from the crown only and 15% with both basal and crown resprouting. Average bark thickness (BT), DBH and relative char height (PCH) were 2.1 cm, 21 cm and 88%, respectively (Table 1).

**Figure 2:**
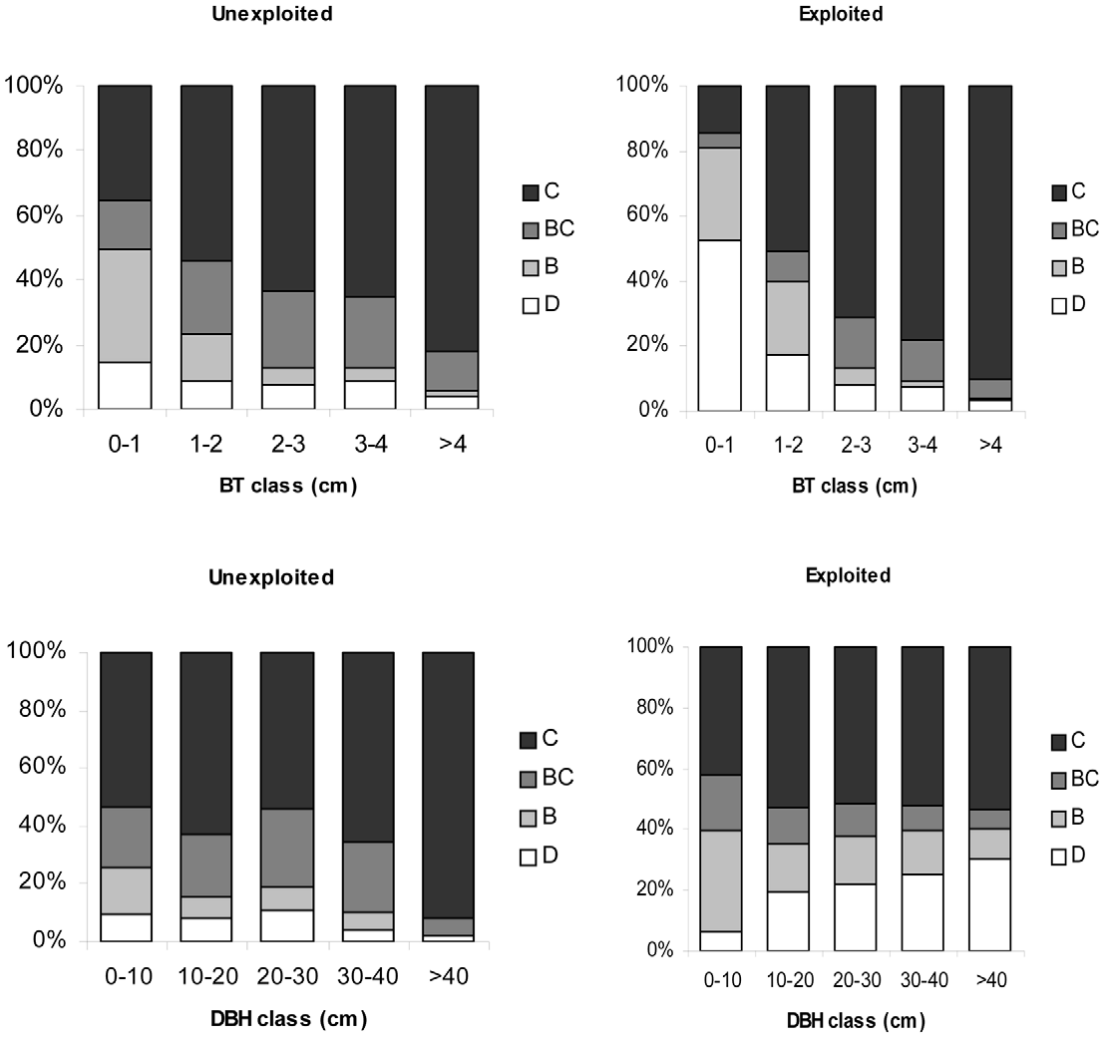
Post-fire *Q. suber* responses. Observed post-fire tree responses (D – dead, B – basal resprouting only, BC – basal and crown resprouting, C – crown resprouting only) as a function of bark exploitation status (exploited, unexploited), bark thickness (BT; figures above) and tree diameter (DBH; figures below). The number of individuals in each class is indicated in the top of the respective bar.

Post-fire *Q. suber* responses were variable between sites. Individual mortality ranged from 0 to 51% and stem mortality from 1 to 64%, as well as individual tree characteristics: the proportion of exploited trees in each site ranged from 0 to 100%; mean BT ranged from 1.2 to 2.9 cm; and mean DBH ranged between 17 and 53 cm. Exploited trees (54% of the total sample) suffered considerably higher mortality (23%) and stem mortality (38%) than the unexploited trees (8% and 19%, respectively), as did thin-barked and larger trees (see Figure 2 and Table S3).

### Cork oak mortality and resprouting

**Figure 3:**
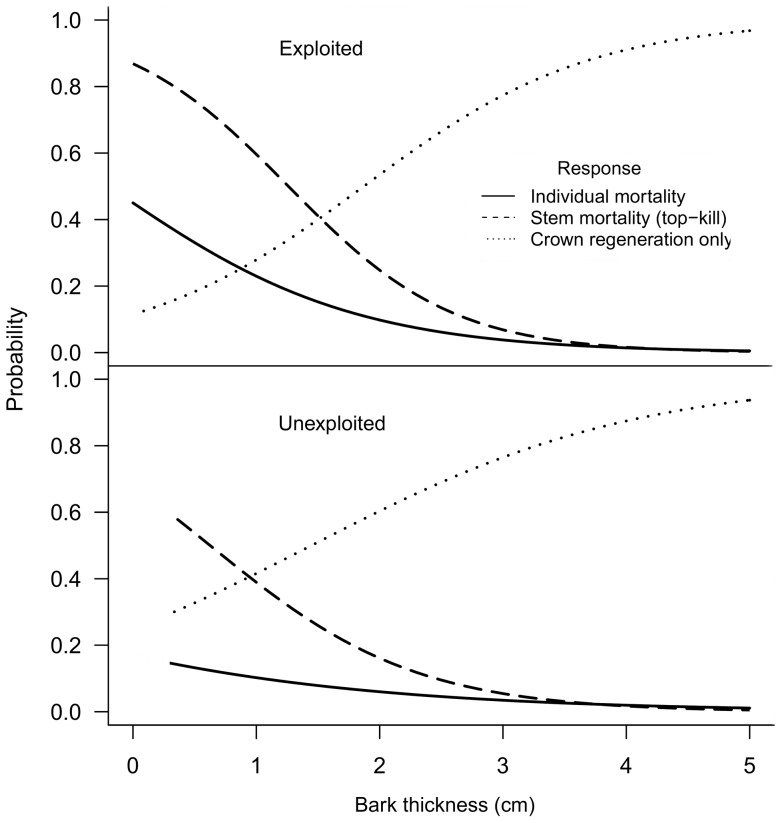
Post-fire *Q. suber* responses as a function of bark thickness and exploitation status. Post-fire *Q. suber* responses as a function of bark thickness and exploitation status based on *overall* models in Table 2 (DBH is held constant at 21 cm, representing the average tree; this size corresponds to the beginning of the productive life for exploited trees).

The obtained mixed models (Table 2 and Table S4) clearly show that BT and bark harvesting are major factors affecting post-fire *Q. suber* responses. On one hand, the thicker the bark the lower the probability of a tree being killed by fire and the higher the probability of regenerating from the crown (Figure 3). On the other hand, exploited trees (Ex) were more likely to be fire-damaged than unexploited trees, regardless of BT, and the interaction between BT and Ex showed that the effect of BT is more important on exploited trees. Tree size (DBH) also affected tree responses; the probability of post-fire tree mortality increases by 40% when DBH increases from 20 to 80 cm.

**Table 2:** Coefficients of the generalized linear mixed-effects models (standard error in brackets) for predicting post-fire *Q. suber* responses.

Post-fire response	Individual mortality (dead)		Stem mortality (top-killed, including dead)		Crown resprouting only	
Level of analysis	Overall	West Iberia	Overall	West Iberia	Overall	West Iberia
Trees (n)	4585	3850	4585	3850	4585	3850
β_0_	−2.01^‡^ (0.30)	−3.25^‡^ (0.44)	0.54 (0.29)	−1.50^‡^ (0.38)	−1.31^‡^ (0.26)	0.56 (0.33)
BT	−0.58^‡^ (0.09)	−0.40^‡^ (0.12)	−1.20^‡^ (0.08)	−1.41^‡^ (0.06)	0.76^‡^ (0.06)	0.69^‡^ (0.07)
Ex (yes)	1.39^‡^ (0.26)	1.66^‡^ (0.32)	1.14^‡^ (0.23)	0.67^‡^ (0.13)	−0.94^‡^ (0.20)	−1.05^‡^ (0.21)
BT* Ex	−0.43^‡^ (0.11)	−0.59^‡^ (0.13)	−0.30^†^ (0.10)	-	0.33^‡^ (0.08)	0.36^‡^ (0.08)
DBH	0.02^‡^ (0.00)	0.03^‡^ (0.00)	0.01^*^ (0.00)	0.02^‡^ (0.00)	−0.01^*^ (0.00)	-
PCH	NA	0.01^†^ (0.00)	NA	0.02^‡^ (0.00)	NA	−0.02^‡^ (0.00)
AUC	0.83	0.82	0.86	0.87	0.82	0.81
R^2^	0.20	0.24	0.37	0.42	0.23	0.27

(1) Model coefficients: β_0_, intercept; BT, bark thickness (cm); Ex, exploited for cork (yes *vs*. no); DBH, diameter at breast height (cm); BT*Ex, interaction between BT and Ex; PCH, maximum bole char height expressed as percentage of tree height (%); NA, means that the variable was not tested because it was not available in all sites; standard error of each coefficient is shown in brackets; (2) Significance of coefficients for the variables refers to the change in explained variance (^*^ *P*<0.05; ^†^ *P*<0.01; ^‡^ *P*<0.001) and for categorical variables refers to the comparison with the first category. (3) Models evaluation: AUC, area under the ROC curve; R^2^, Nagelkerke R^2^. SHAPE.

*Q*. *suber* vulnerability to fire also increased with increasing char height percentage (PCH). PCH had a stronger effect on stem mortality than on individual mortality (up to 40% higher probability of stem mortality for trees with 100% PCH than for trees with 25% PCH), and was similar for exploited and unexploited trees.

Both the *overall* and *west Iberia* models produced similar predictions. Tree responses represented in Figure 3 correspond to relatively young trees under moderate to severe fire conditions. However, the fire impacts can be much stronger for larger trees with high proportion of char height. For example, the probability of mortality is very high (92%) for recently debarked and totally charred (100% PCH) large trees (with 100 cm DBH).

The obtained models (Table 2) performed well, with ROC curves (AUC) indicating 81 to 87 percent agreement between predicted probabilities and observed outcomes. The models predicting top-kill (stem mortality + tree mortality) were those with better performance and higher explained variance. The sensitivity analysis considering the potential error in bark thickness measurements yielded very similar models (Text S1).

The random (site-level) effects in post-fire *Q. suber* responses were related to fire season and aspect. Early season fires had significantly higher coefficients (*P* = 0.02), suggesting that tree mortality was higher in sites burned in early summer than in those burned in late summer or winter. There was also a positive correlation between site coefficients in the stem mortality models and the proportion of trees in unfavourable aspects on each site (*r* = 0.51, *P* = 0.02), confirming the detrimental effect of southern aspects on tree stem survival. For the remaining environmental variables no significant associations with site-level effects were found in any response type.

## Discussion

### Influence of bark thickness and bark exploitation

Our results clearly indicate that bark thickness (BT) is a major determinant of the post-fire responses of *Q. suber*, and this is especially relevant in trees that are subjected to bark exploitation. Tree vulnerability to fire significantly decreases with increasing BT until bark is about 4 cm thick. Trees with bark thicker than 3–4 cm are well protected against heat injury and are very unlikely to die or to suffer stem mortality, i.e. they will likely resprout from the crown. Bark thickness is a key fire resistance factor for many other tree species worldwide [14]–[15], [44]–[46]. Our results suggest that the probability of top-kill in *Q. suber* is considerably lower than in other Mediterranean broadleaves [30], even when bark is thinner than 3 cm. This can be explained by the low thermal conductivity properties of cork, which makes this material an excellent heat insulator [47]. The sensitivity analysis considering the potential error in BT measurements due to variations with time (see methods) yielded very similar models, strongly suggesting that this potential bias did not influence the post-fire response patterns obtained.

Cork harvesting does not only drastically reduces bark thickness but it has additional effects. That is, in addition to BT, cork exploitation *per se* has a significant influence on tree resistance to fire, such that unexploited trees showed significantly less mortality and stem death than exploited trees, even for trees with the same bark thickness. In fact, debarking is a stress factor for trees and has been associated to vigour loss (e.g. [12]). Cork is usually extracted from stem and thicker branches by manually cutting with an axe along vertical and horizontal lines around the tree perimeter, and subsequently pulling out large cork planks. Such operation is performed during the period of periderm activity, when it is relatively easy to separate the cork layer at the level of phellogenic active zone (i.e. cork cambium) without damaging the underlying phloem and vascular cambium [3], [12]. This induces the formation of a new phellogen and cork regrowth, in such a way that cork oak trees can usually withstand repeated bark harvesting during their lives [3], [12]. However, cork harvesting has immediate direct negative physiological effects on exploited individuals leading to considerable water losses through the stripped trunk surface. Additionally, stoma close quickly in the hours following debarking leading to interruption of the nutritional functions, which only return to normal after 24–30 days [3], [12], [48]. During this period the traumatic phellogen is formed and some layers of cork cells are produced thereby protecting the active phloem from further water losses. This process requires a very high consumption of reserves, leading to a decrease in the vascular cambium activity and a stop in wood growth during this period [3], [12]. Additionally, the wounds caused by mechanical damages to the inner bark and vascular cambium during the cork harvesting operations [3] can also be associated with loss of tree vigour [49]. In our database, we had information about the presence of stem wounds in 36% of the sampled trees and verified that 63.1% of all exploited trees had stem wounds, contrasting with only 2.8% among unexploited trees (*χ*2 = 596.246, *P*<0.001). We also verified that the presence of wounds is a better predictor of tree mortality than the exploitation status, suggesting that stem wounds are probably one of the main factors decreasing tree resistance to fire. Wounded trees are likely more vulnerable because bark is very thin or absent near wounds, making the trunk more sensitive to heat and to other agents, including beetle attacks and fungi infections [50]–[52]. Wounding is also likely to reduce tree vigour, both because of the energy resources that trees need for cicatrisation, and because the death of active xylem decreases the rate of nutrient and water absorption [53].

### Influence of tree size and fire severity

Our results indicate that larger trees (with higher DBH) are more likely to die or to suffer stem mortality than smaller ones, suggesting that the high maintenance cost of large trees may be relevant for their resprouting failure. In fact, basal resprouting ability has been reported to consistently decrease with tree DBH and age in several other oaks that are not recurrently debarked [33]. Diameter was somewhat related to the exploitation status (r = 0.46), with unexploited trees being often smaller than exploited trees (mean DBH of 14 and 27 cm, respectively). On the other hand, higher susceptibility of the larger exploited individuals could be expected. These trees have been probably debarked more often during their lifetime and were probably subjected more often to poor management practices [12], [28]. Also, larger trees are often debarked up to the branches as well (while smaller trees are debarked in the stem only), potentially exposing a larger surface of the tree to fire damage. However the lack of a significant interaction between DBH and Ex suggests that DBH exerts a similar effect on exploited and unexploited trees.

*Q*. *suber* vulnerability to fire significantly increased with increasing tree char height (PCH), as expected. However, the limited importance of PCH in the models might be because of the relatively low variability of this parameter (mean  = 88%; median  = 100%) or because this metric is not the most appropriate for describing the level of injury; in such cases, other variables (e.g. char depth) could eventually be better indicators of injury.

### Environmental correlates of between-site variations

Trees located in sites that burned earlier in the summer were more likely to die than those burning later. Other studies reported that plants are more susceptible to fire when they are flowering, actively growing, or when carbohydrate reserves are relatively low [33], [54]. In fact some tree species appear more susceptible to early growing season burns than to late growing season burns [33], [54]. This has been associated to the fact that both dead and live tissues moisture content is often higher in the early growing season, increasing thermal conductivity, heat exposure and the likelihood of necrosis in vulnerable tissues (e.g. elongating meristems). Additionally, carbohydrate reserves are also lower early in the growing season [33], [54]. Although *Q. suber* is an evergreen species, spring and early summer are its main growing and flowering periods [12], [55], thus seasonal physiological variation may explain the higher mortality of individuals in early summer fires.

Trees located in sites with a higher proportion of south-facing aspects were also more vulnerable to fire. In the Mediterranean, south-facing slopes receive higher solar radiation, which increases temperature and reduces water availability to plants [34], [56]. These slopes usually have less vegetation cover and a thinner soil layer, being more vulnerable to soil erosion [57]. Additionally, some of the more important diseases and insects affecting *Q. suber* have been reported to have higher incidence on south-facing slopes [58], [59]. All these unfavourable conditions are likely to increase tree stress and consequently increase vulnerability to wildfires.

### Implications for management and conservation

Cork is a valuable natural resource and cork harvesting is currently the main reason for managers to maintain *Q. suber* ecosystems. However, bark harvesting is also a major factor contributing to increased tree vulnerability to fire damage. Thus, although *Q. suber* is usually recognized as one of the most fire-resistant and resilient Mediterranean tree species, wildfires can cause major economic and ecological impacts in ecosystems managed for cork production.

The vulnerability of exploited trees is at its highest level immediately after bark harvesting (up to nearly 100% probability of mortality), and then it decreases with time as bark regrows until cork is 3–4 cm thick, which is usually attained by the end of the cork production cycle (9 to 15 years). Thus, during most of the time (particularly during the first half of the cycle) the exploited trees are much more vulnerable to fire than unexploited trees due to a longer period with thin bark. Therefore managers need to be aware of this to assure preventive measures. Furthermore, several studies indicate a current and future trend of increasing wildfire occurrence and severity due to changes in land management and climate [20], [24], [25], [60], which will contribute to threaten cork oak ecosystems in the Mediterranean Basin. Climate change is also predicted to increase water stress [61] and to favour the spread of oak diseases in this region [62], decreasing the vigour of trees, and consequently further increasing their susceptibility to fire.

A strong negative economic impact is expected in burned *Q. suber* stands, both because the charred bark looses its value, and bark productivity decreases. The minimum time required to start harvesting good quality cork (i.e. cork used to produce good quality stoppers) after fire is about 40 years for trees that died and need to be replaced [3], 30 years for the surviving trees with stem mortality and 10 years for trees with good crown regeneration [63]. The models presented can be used to help managers predicting post-fire tree responses, and thus improving their ability to estimate the impacts from fire. These impacts may include changes in cork production, carbon stocks, wildlife habitat, water retention and soil erosion. Models can also help planning post-fire management activities such as coppicing the more severely damaged trees [29] and assisting natural regeneration.

Several alternative or complementary actions can be implemented in order to reduce fire damage in managed *Q. suber* stands [64]. Treating surface fuels just before debarking (i.e. every 9 to 15 years) and promoting less flammable species in the understorey, would reduce fire intensity, hence char height, and could be an effective mitigation action to avoid severe fires particularly during the first half of the cork production cycle when trees are more vulnerable. An additional fuel treatment in the middle of the cycle could be recommended in high productivity sites where burn probability is higher. Fuel treatments need to be done with care to avoid root damage, and the biodiversity implications need to be considered. Careful management of cork harvesting activities could also decrease tree vulnerability to fire. Stem wounds, which are often inflicted to trees during bark harvesting operations, would be avoided e.g. by employing skilled workers or using automatic equipment for harvesting [63]. Before debarking, trees should be allowed time to recover from other stressing events, such as branch pruning, insect outbreaks, droughts or fires [18], [64]. Other measures could include debarking coexisting trees of a given stand in different years (reducing the overall forest vulnerability) or increasing the length of the cork harvesting cycle and consequently increasing the time during which the trees have thicker bark and are better protected against fire injury. Such extension of the cork production cycle would not necessarily imply lower economic income [12]. Since cork is the main economical income from these forests, stopping bark exploitation might be unrealistic in most cases. However, in fire-prone areas where conservation is the main objective, this would likely be the most effective option to increase ecosystem resilience to fire. The valorisation of many other services provided by cork oak forests (e.g. [8]) could create economic incentives to decrease the bark-exploitation dependency of these systems in the future.

## Supporting Information

Table S1 **Main characteristics of the 22 study sites.**
(DOC)

Table S2 **List of the main tree characteristics by site.**
(DOC)

Table S3 **List of the main post-fire tree responses by site.**
(DOC)

Table S4 **Summary of the sequential ANOVA for each post-fire response model.**
(DOC)

Text S1 **Sensitivity analysis accounting for potential bark thickness measurement constraints.**
(DOC)
