# Supplementary material for: Cork Oak Vulnerability to Fire: The Role of Bark Harvesting, Tree Characteristics and Abiotic Factors
Source: PLoS One. 2012 Jun 28;7(6):e39810. doi: 10.1371/journal.pone.0039810 (PMC3386235; doi:10.1371/journal.pone.0039810)
Supplement: Table S1 — Main characteristics of the 22 study sites. (DOC) [file pone.0039810.s001.doc]

Table S1. List of the 22 studied wildfire sites in Portugal, Spain and France, including some site characteristics.

| General location | Country | Site | Region | Fire date | Trees (n) | Plots (n) | Elevation (m) | Prec. (mm) | Temp.  (ºC) | Years SF |
| --- | --- | --- | --- | --- | --- | --- | --- | --- | --- | --- |
| West Iberia | Portugal | Avidagos | 159 | 3 Aug. 2003 | 150 | 5 | 400 | 550 | 15.5 | 2 |
|  |  | Barrancos | 159 | 15 July 2006 | 78 | 6 | 313 | 550 | 16.8 | 2 |
|  |  | Cedães | 159 | 19 Aug. 2005 | 88 | 3 | 550 | 550 | 15.5 | 2 |
|  |  | Évora | 159 | 7 Sep. 2006 | 120 | 4 | 321. | 750 | 15.5 | 1 |
|  |  | Franco | 159 | 15 July 2005 | 120 | 4 | 415 | 550 | 15.5 | 2 |
|  |  | Freixiel | 159 | 4 Aug. 2002 | 150 | 5 | 500 | 900 | 13.8 | 3 |
|  |  | Mirandela | 159 | 31 July 2006 | 352 | 12 | 300 | 550 | 15.5 | 1 |
|  |  | Agolada | 168 | 29 Aug. 2006 | 30 | 1 | 64 | 650 | 15.5 | 1 |
|  |  | Agroal | 168 | 4 Sep. 2006 | 24 | 7 | 163 | 750 | 16.8 | 1 |
|  |  | Caldeirão^a^ | 168 | 26 July 2004 | 1132 | 40 | 430 | 900 | 16.6 | 1 |
|  |  | Frazão | 168 | 19 June 2003 | 300 | 10 | 107 | 650 | 15.5 | 4 |
|  |  | Mafra^b^ | 168 | 11 Sep. 2003 | 326 | 12 | 162 | 750 | 13.8 | 4 |
|  |  | Portel | 168 | 3 Aug. 2005 | 300 | 10 | 325 | 650 | 16.8 | 2 |
|  |  | Raposa | 168 | 1 Aug. 2003 | 305 | 11 | 42 | 650 | 15.5 | 4 |
|  |  | V. Covo | 168 | 30 June 2004 | 143 | 5 | 77 | 650 | 15.5 | 3 |
|  |  | V. Florido | 168 | 6 Aug. 2006 | 33 | 5 | 333 | 1100 | 16.8 | 1 |
|  | Spain | Cañaveral | 159 | 1 Aug. 2003 | 29 | 3 | 470 | 715 | 15.9 | 1 |
|  |  | Carmonita | 159 | 2 Aug. 2003 | 58 | 6 | 460 | 650 | 15.5 | 1 |
|  |  | V. Alcantara | 168 | 1 Aug. 2003 | 112 | 11 | 485 | 550 | 15.6 | 1 |
| East Iberia and South France |  | Espadà | 162 | 31 Jan. 2003 | 269 | 20 | 606 | 558 | 13.4 | 1 |
|  |  | Girona^c^ | 162 | 10 Aug. 1994 | 115 | 4 | 408 | 820 | 17.0 | 2 |
|  | France | Maures massif ^d^ | 162 | 29 Jul. 2003 | 351 | 19 | 650 | 1050 | 11.5 | 3 |

(1) General geographic location; Country; Site, name of the study site; Region, ecological region code (EEA 2003; codes: 168 - Southwest Iberian Mediterranean sclerophyllous and mixed forests; 159 - Iberian sclerophyllous and semi-deciduous forests; 162 - Northeastern Spain & Southern France Mediterranean); Fire starting date; Trees, number of sampled trees in each site; Plots, number of sampled plots; Elevation, mean elevation of the site; Prec., mean precipitation; Temp., mean temperature; Years SF, number of years between the fire and the sampling. (2) References: ^a^ Moreira et al. 2007, 2009; Catry et al. 2009; ^b^ Catry et al. 2010; ^c^ Pausas 1997; ^d^ Curt et al. 2010; (data from the remaining sites have been described in technical reports and graduation thesis).
