# Supplementary material for: Cork Oak Vulnerability to Fire: The Role of Bark Harvesting, Tree Characteristics and Abiotic Factors
Source: PLoS One. 2012 Jun 28;7(6):e39810. doi: 10.1371/journal.pone.0039810 (PMC3386235; doi:10.1371/journal.pone.0039810)
Supplement: Table S2 — List of the main tree characteristics by site. (DOC) [file pone.0039810.s002.doc]

Table S2. List of the 22 fire sites and main tree characteristics.

| General location | Country | Site | Sampled trees (n) | | | DBH (cm) | | | BT (cm) | | | PCH (%) | | |
| --- | --- | --- | --- | --- | --- | --- | --- | --- | --- | --- | --- | --- | --- | --- |
|  |  |  | Total | Ex. | UEx. | Total | Ex. | UEx. | Total | Ex. | UEx. | Total | Ex. | UEx. |
| West Iberia | Portugal | Avidagos | 150 | 91 | 59 | 35 (15) | 37.4 (13.5) | 17.6 (7.6) | 2.6 (1.0) | 2.3 (1.0) | 3.0 (0.9) | 84 | 79 | 93 |
|  |  | Barrancos | 78 | 44 | 34 | 28 (11) | 30.0 (7.7) | 15.1 (6.2) | 2.2 (1.0) | 2.4 (0.9) | 2.0 (1.1) | 93 | 94 | 92 |
|  |  | Cedães | 88 | 18 | 70 | 19 (14) | 26.8 (10.6) | 11.9 (12.0) | 1.8 (0.8) | 2.0 (1.0) | 1.7 (0.8) | 98 | 100 | 97 |
|  |  | Évora | 120 | 52 | 68 | 29 (18) | 37.1 (18.5) | 13.5 (6.0) | 2.8 (0.6) | 2.9 (0.5) | 2.8 (0.6) | 100 | 100 | 100 |
|  |  | Franco | 120 | 63 | 57 | 26 (18) | 33.4 (19.5) | 13.1 (6.1) | 1.2 (0.6) | 1.1 (0.7) | 1.3 (0.5) | 83 | 74 | 92 |
|  |  | Freixiel | 150 | 63 | 87 | 30 (12) | 35.2 (9.2) | 18.6 (6.1) | 2.4 (0.8) | 2.5 (1.0) | 2.3 (0.5) | 99 | 100 | 99 |
|  |  | Mirandela | 352 | 132 | 220 | 23 (13) | 30.3 (10.6) | 12.4 (8.2) | 1.4 (0.7) | 1.2 (0.8) | 1.5 (0.7) | 91 | 85 | 96 |
|  |  | Agolada | 30 | 22 | 8 | 30 (13) | 28.5 (10.2) | 11.3 (4.5) | 2.9 (0.6) | 2.9 (0.6) | 2.7 (0.6) | 94 | 93 | 96 |
|  |  | Agroal | 24 | 0 | 24 | 17 (6) | - | 12.5 (4.7) | 2.1 (0.7) | - | 2.1 (0.7) | 97 | - | 97 |
|  |  | Caldeirão | 1132 | 840 | 292 | 27 (12) | 26.0 (11.9) | 10.7 (3.5) | 2.6 (1.2) | 2.4 (1.3) | 2.9 (0.8) | 78 | 79 | 76 |
|  |  | Frazão | 300 | 272 | 28 | 27 (9) | 25.7 (8.7) | 9.9 (4.8) | 1.4 (1.2) | 1.2 (1.1) | 2.8 (0.5) | 91 | 90 | 96 |
|  |  | Mafra | 326 | 195 | 131 | 28 (18) | 20.2 (7.6) | 24.1 (18.5) | 2.8 (1.7) | 1.8 (0.8) | 3.5 (1.8) | 91 | 97 | 87 |
|  |  | Portel | 300 | 199 | 101 | 23 (7) | 22.0 (6.8) | 15.2 (5.8) | 1.7 (1.1) | 1.3 (1.1) | 2.3 (0.9) | 94 | 95 | 92 |
|  |  | Raposa | 305 | 161 | 144 | 19 (9) | 20.6 (10.4) | 8.5 (2.2) | 1.9 (0.8) | 1.6 (0.9) | 2.3 (0.5) | 97 | 97 | 97 |
|  |  | V. Covo | 143 | 115 | 28 | 23 (10) | 20.9 (9.6) | 9.5 (3.0) | 2.2 (0.6) | 2.2 (0.6) | 2.2 (0.4) | 83 | 80 | 97 |
|  |  | V. Florido | 33 | 22 | 11 | 23 (8) | 22.4 (7.1) | 12.4 (3.0) | 1.8 (1.1) | 1.7 (1.3) | 2.1 (0.7) | 100 | 100 | 100 |
|  | Spain | Cañaveral | 29 | 29 | 0 | 53 (24) | 53 (23.7) | - | 2.5 (1.0) | 2.5 (1.0) | - | 56 | 56 | - |
|  |  | Carmonita | 58 | 20 | 38 | 19 (15) | 34.3 (16.7) | 10.4 (4.7) | 2.0 (1.0) | 1.5 (0.8) | 2.3 (1.0) | 90 | 83 | 94 |
|  |  | V. Alcantara | 112 | 78 | 34 | 39 (26) | 49.7 (23.5) | 13.0 (8.0) | 2.1 (1.2) | 2.1 (1.2) | 2.3 (1.1) | 85 | 83 | 91 |
| East Iberia and South France |  | Espada | 269 | 94 | 175 | 19 (9) | 24.5 (7.3) | 10.4 (3.3) | 1.6 (0.6) | 1.8 (0.6) | 1.4 (0.6) | - | - | - |
|  |  | Girona | 115 | 40 | 75 | 19 (11) | 24.6 (6.8) | 6.8 (3.4) | 2.3 (0.9) | 3.1 (0.7) | 1.7 (0.7) | - | - | - |
|  | France | Maures massif | 351 | 0 | 351 | 25 (17) | - | 2.6 (1.1) | 2.6 (1.1) | - | 19.5 (15.5) | 88 | - | - |
| Mediterranean |  | All | 4585 | 2486 | 2099 | 21 (14) | 27.2 (13.4) | 14.3 (11.0) | 2.1 (1.2) | 2.0 (1.2) | 2.3 (1.1) | 88 | 86 | 91 |

(1) General location; Country; Fire site, name of the study site; Sampled trees, total number of sampled trees; DBH, mean (SD) diameter at breast height; BT, mean (SD) bark thickness; PCH, mean maximum bole char height expressed as percentage of tree height; Ex. and UEx. represent the exploited and unexploited trees, respectively.
