# Supplementary material for: Cork Oak Vulnerability to Fire: The Role of Bark Harvesting, Tree Characteristics and Abiotic Factors
Source: PLoS One. 2012 Jun 28;7(6):e39810. doi: 10.1371/journal.pone.0039810 (PMC3386235; doi:10.1371/journal.pone.0039810)
Supplement: Table S3 — List of the main post-fire tree responses by site. (DOC) [file pone.0039810.s003.doc]

Table S3. List of the 22 fire sites and main post-fire responses: individual mortality and top-kill.

| General location | Country | Site | Trees (n) | Mortality (%) | | | Top kill (%) | | | |
| --- | --- | --- | --- | --- | --- | --- | --- | --- | --- | --- |
|  |  |  |  | Total | Ex. | UEx. | | Total | Ex. | UEx |
| West Iberia | Portugal | Avidagos | 150 | 8.0 | 12.1 | 1.7 | | 28.7 | 42.9 | 6.8 |
|  |  | Barrancos | 78 | 10.3 | 13.6 | 5.9 | | 15.4 | 20.5 | 8.8 |
|  |  | Cedães | 88 | 0.0 | 0.0 | 0.0 | | 1.1 | 0.0 | 1.4 |
|  |  | Évora | 120 | 5.8 | 7.7 | 4.4 | | 14.2 | 11.5 | 16.2 |
|  |  | Franco | 120 | 12.6 | 17.5 | 7.1 | | 46.2 | 57.1 | 33.9 |
|  |  | Freixiel | 150 | 6.0 | 9.5 | 3.4 | | 11.3 | 22.2 | 3.4 |
|  |  | Mirandela | 352 | 13.6 | 28.8 | 3.9 | | 26.0 | 34.1 | 20.9 |
|  |  | Agolada | 30 | 10.0 | 4.5 | 25.0 | | 10.0 | 4.5 | 25.0 |
|  |  | Agroal | 24 | 0.0 | - | 0.0 | | 8.3 | - | 8.3 |
|  |  | Caldeirão | 1132 | 15.9 | 18.0 | 9.9 | | 21.3 | 24.3 | 12.7 |
|  |  | Frazão | 300 | 51.0 | 56.3 | 0.0 | | 63.7 | 70.2 | 0.0 |
|  |  | Mafra | 326 | 8.3 | 15.3 | 3.6 | | 33.7 | 66.4 | 11.8 |
|  |  | Portel | 300 | 32.0 | 42.2 | 11.9 | | 52.3 | 70.4 | 16.8 |
|  |  | Raposa | 305 | 17.0 | 24.8 | 8.3 | | 40.3 | 60.2 | 18.1 |
|  |  | V. Covo | 143 | 5.6 | 6.1 | 3.6 | | 14.0 | 16.5 | 3.6 |
|  |  | V. Florido | 33 | 9.1 | 13.6 | 0.0 | | 21.2 | 31.8 | 0.0 |
|  | Spain | Cañaveral | 29 | 17.2 | 17.2 | - | | 20.7 | 20.7 | - |
|  |  | Carmonita | 58 | 13.8 | 40.0 | 0.0 | | 39.7 | 60.0 | 28.9 |
|  |  | V. Alcantara | 112 | 17.0 | 23.1 | 2.9 | | 28.6 | 33.3 | 17.6 |
| East Iberia and South France |  | Espada | 269 | 0.7 | 0.0 | 1.1 | | 4.1 | 0.0 | 6.3 |
|  |  | Girona | 115 | 0.9 | 0.0 | 1.3 | | 32.2 | 0.0 | 49.3 |
|  | France | Maures massif | 351 | 24.6 | - | 24.6 | | 39.2 | - | 39.2 |
| Mediterranean |  | All | 4585 | 16.2 | 22.8 | 8.5 | | 29.3 | 37.8 | 19.3 |

(1) General location; Country; Fire site, name of the study site; Trees, number of sampled trees; Mortality, percentage of dead trees; Top kill, percentage of trees with stem mortality; Ex. and UEx represent the exploited and unexploited trees, respectively.
