# Supplementary material for: Cork Oak Vulnerability to Fire: The Role of Bark Harvesting, Tree Characteristics and Abiotic Factors
Source: PLoS One. 2012 Jun 28;7(6):e39810. doi: 10.1371/journal.pone.0039810 (PMC3386235; doi:10.1371/journal.pone.0039810)
Supplement: Table S4 — Summary of the sequential ANOVA for each post-fire response model. (DOC) [file pone.0039810.s004.doc]

Table S4. Summary of the sequential ANOVA for each post-fire response model, applied to the variables previously selected by the stepwise procedure (variables are added sequentially in the order of their contribution to the remaining explained deviance).

| Models* | Overall (n=4585 trees) | | | | | West Iberia (n=3850 trees) | | | | | |
| --- | --- | --- | --- | --- | --- | --- | --- | --- | --- | --- | --- |
|  | Df | AIC | LogLik | *χ*^2^ | *P-*value |  | Df | AIC | LogLik | *χ*^2^ | *P-*value |
| Individual mortality | | | | | | Individual mortality | | | | | |
| Null model | 2 | 3673.5 | -1834.8 | - | - | Null model | 2 | 3223.7 | -1609.8 | - | - |
| +BT | 3 | 3228.4 | -1611.2 | 447.1 | <0.0001 | +BT | 3 | 2792.6 | -1393.3 | 433.1 | <0.0001 |
| +DBH | 4 | 3162.3 | -1577.2 | 681 | <0.0001 | +DBH | 4 | 2704.3 | -1348.2 | 90.3 | <0.0001 |
| +Ex | 5 | 3150.5 | -1570.2 | 30.8 | <0.0001 | +Ex | 5 | 2697.2 | -1343.6 | 9.1 | 0.0025 |
| +BT*Ex | 6 | 3136.7 | -1562.3 | 15.8 | <0.0001 | +BT*Ex | 6 | 2681.3 | -1334.7 | 17.9 | <0.0001 |
| - | - | - | - | - | - | +PCH | 7 | 2673.7 | -1329.8 | 9.6 | 0.0019 |
| Stem mortality (top-kill) | | | | | | Stem mortality (top-kill) | | | | | |
| Null model | 2 | 5076.9 | -2536.5 | - | - | Null model | 2 | 4354.6 | -2175.3 | - | - |
| +BT | 3 | 3860.3 | -1927.1 | 1218.7 | <0.0001 | +BT | 3 | 3209.1 | -1601.5 | 1147.5 | <0.0001 |
| +Ex | 4 | 3808.7 | -1900.3 | 53.6 | <0.0001 | +Ex | 4 | 3146.1 | -1569.0 | 65.0 | <0.0001 |
| +BT*Ex | 5 | 3799.0 | -1894.5 | 11.6 | 0.0006 | +PCH | 5 | 3080.7 | -1535.4 | 67.4 | <0.0001 |
| +DBH | 6 | 3796.3 | -1892.2 | 4.7 | 0.0299 | +DBH | 6 | 3062.8 | -1525.4 | 20.0 | <0.0001 |
| Crown regeneration only | | | | | | Crown regeneration only | | | | | |
| Null model | 2 | 5702.4 | -2849.2 | - | - | Null model | 2 | 4972.6 | -2484.3 | - | - |
| +BT | 3 | 4890.8 | -2442.4 | 813.6 | <0.0001 | +BT | 3 | 4234.7 | -2114.3 | 739.9 | <0.0001 |
| +Ex | 4 | 4890.5 | -2441.3 | 2.3 | 0.1307 | +Ex | 4 | 4231.7 | -2111.9 | 4.9 | 0.0265 |
| +BT*Ex | 5 | 4878.6 | -2434.3 | 13.9 | 0.0002 | +BT*Ex | 5 | 4221.3 | -2105.6 | 12.5 | 0.0004 |
| +DBH | 6 | 4875.5 | -2431.8 | 5.1 | 0.0237 | +PCH | 6 | 4152.0 | -2070.0 | 71.3 | <0.0001 |

*For each post-fire response type: (1) Variables: BT, bark thickness (cm); Ex, exploited for cork (yes *vs*. no); DBH, diameter at breast height (cm); BT*Ex, interaction between BT and Ex; PCH, maximum bole char height expressed as percentage of tree height (%); NA, means that the variable was not tested because it was not available in all sites; (2) Model statistics: Df, degrees of freedom; AIC, Akaike information criteria; LogLik, log-likelihood; *χ*^2^, chi-square; *P-*value, significance level.
