# Supplementary material for: Cork Oak Vulnerability to Fire: The Role of Bark Harvesting, Tree Characteristics and Abiotic Factors
Source: PLoS One. 2012 Jun 28;7(6):e39810. doi: 10.1371/journal.pone.0039810 (PMC3386235; doi:10.1371/journal.pone.0039810)
Supplement: Text S1 — Sensitivity analysis accounting for potential bark thickness measurement constraints. (DOC) [file pone.0039810.s005.doc]

**Sensitivity analysis accounting for potential bark thickness measurement constraints**

*Correction of bark thickness estimates*

In order to evaluate to what extent the potential problems of BT measurement under- or overestimation could be affecting our results, we created a new variable consisting of an estimated BT at the time of the fire (EBT). This was derived from our field measurements, but with the following correction factors:

(i) Correcting for the potential loss of bark caused by the fire: We found no information on the potential loss of cork thickness after fire. Cork is a very good insulation material, with low flammability, which does not burn easily; however, in severe fires some consumption of the external layers can occur. Usually the scorched cork depth seems to represent 20 to 25% of the total bark thickness [49], but there is no evidence at all that this scorched bark will be lost from the tree. Given that many of our trees had a relatively thin bark, we increased BT in 2 mm to all trees, to take into account the external cork layers that could have been consumed during the fire.

(ii) Correcting for the potential growth of bark after fire: we decreased BT, accounting for potential bark growth that might have occurred between the fire and BT measurements. Since we have not found any specific information in the literature about cork growth after fire, we used as reference a mean annual cork growth value for unburned trees (exploited trees = 3.3 mm and unexploited trees = 1.5 mm; cork growth data from 65 sites in Portugal, Spain and France - based on [3]). However, we introduced a correction factor because post-fire bark growth is not expected to be the same on unburned and burned trees (Table 1). In spite of the lack of information about the impact of fire on cork growth, it is known that several stressing factors such as insect attacks, wounding or adverse weather conditions can cause the suspension or the reduction of bark growth in the following years [3,12,49]. For example crown defoliation caused by insects (Lymantria dispar) was reported to suddenly reduce cork oak bark growth, maintaining its influence during the subsequent years [12]. Thus it is likely that a defoliation caused by fire can have the same type of consequences. Considering that in 90% of the sampled trees the maximum char height was more than 55% of the tree height, (and 100% PCH in 60% of the trees), we can assume that almost all trees suffered total crown defoliation. Additionally to defoliation, fire is likely to cause other types of damages such as branch, stem or root injury that will contribute to weaken the physiological status of the burned trees and delay bark growth. For example some authors [49] reported a negative effect of stripping wounds on cork growth (13-16% reduction), particularly noticed in the two first years after wounding. Although the trees have energy reserves to recover, these reserves will be primarily used to cover the vital needs for survival, namely to rebuild the crown or for wound cicatrisation [e.g. 62], thus bark growth will not be a priority. Considering all these issues, and in order to try to keep the correction as realistic and simple as possible, we opted to introduce a reduction factor which started by 100% (i.e. assuming no bark growth during the first year after fire), and decreased progressively at 25% intervals (see Table 1). This correction was only applied to the trees with stem survival (71% of all trees), as for the others the cambium died and no additional cork growth is expected to have occurred after fire.

Table 1. Bark thickness corrections applied to burned cork oak trees with stem survival, as a function of time between fire and tree assessment (TSF). Based on mean annual cork growth of unburned trees: unexploited = 1.5 mm; exploited = 3.3 mm (Pereira et al, 2007).

| Time after fire (years) | | 1 | 2 | 3 | 4 |
| --- | --- | --- | --- | --- | --- |
| Annual bark growth reduction (%) | | 100% | 75% | 50% | 25% |
| Cumulative growth reduction (mm) | Unexploited trees | 0 | 0.4 | 1.1 | 2.3 |
|  | Exploited trees | 0 | 0.8 | 2.5 | 5.0 |

*Models comparison*

After the BT correction, new models were fitted and compared with the ones with the original BT measurements, to analyse if the variables selected or their importance changed. Results show that both the variables selected and their signs did not changed and the coefficients registered only slight variations (Table 2 and Fig. 1). Models performance (evaluated through AUC and R^2^) also remained practically unchanged (Table 2). Based on these results, we consider that the potential problem of post-fire BT under- or overestimation is not important in this particular case, and can be accepted.

Table 2. Coefficients of the overall generalized linear mixed-effects models (standard error in brackets) for predicting post-fire *Q. suber* responses. Comparison between the models using the original versus corrected bark thickness.

| Post-fire response | Individual mortality  (dead) | | Stem mortality  (top-killed, including dead) | | Crown resprouting only | |
| --- | --- | --- | --- | --- | --- | --- |
| Level of analysis | Original model (1) | Corrected model (2) | Original model (1) | Corrected model (2) | Original model (1) | Corrected model (2) |
| Trees (n) | 4585 | 4585 | 4585 | 4585 | 4585 | 4585 |
| β_0_ | -2.01^‡^ (0.30) | -1.94^‡^ (0.30) | 0.54 (0.29) | -1.62^*^ (0.28) | -1.31^‡^ (0.26) | -1.35^‡^ (0.26) |
| BT | -0.58^‡^ (0.09) | -0.57^‡^ (0.09) | -1.20^‡^ (0.08) | -1.15^‡^ (0.08) | 0.76^‡^ (0.06) | 0.74^‡^ (0.06) |
| Ex (yes) | 1.39^‡^ (0.26) | 1.37^‡^ (0.27) | 1.14^‡^ (0.23) | 1.22^‡^ (0.24) | -0.94^‡^ (0.20) | -1.02^‡^ (0.21) |
| BT* Ex | -0.43^‡^ (0.11) | -0.44^‡^ (0.11) | -0.30^†^ (0.10) | -0.41^‡^ (0.10) | 0.33^‡^ (0.08) | 0.39^‡^ (0.08) |
| DBH | 0.02^‡^ (0.00) | 0.02^‡^ (0.00) | 0.01^*^ (0.00) | 0.01^*^ (0.00) | -0.01^*^ (0.00) | -0.01^*^ (0.00) |
| AUC | 0.83 | 0.83 | 0.86 | 0.86 | 0.82 | 0.82 |
| R^2^ | 0.20 | 0.20 | 0.37 | 0.37 | 0.23 | 0.24 |

1. Model coefficients: β0, intercept; BT, bark thickness (cm); Ex, exploited for cork (yes vs. no); DBH, diameter at breast height (cm); BT*Ex, interaction between BT and Ex; standard error of each coefficient is shown in brackets; (2) Significance of coefficients for the variables refers to the change in explained variance (* *P*<0.05; † *P*<0.01; ‡ *P*<0.001) and for categorical variables refers to the comparison with the first category. (3) Models evaluation: AUC, area under the ROC curve; R^2^, Nagelkerke R^2^.


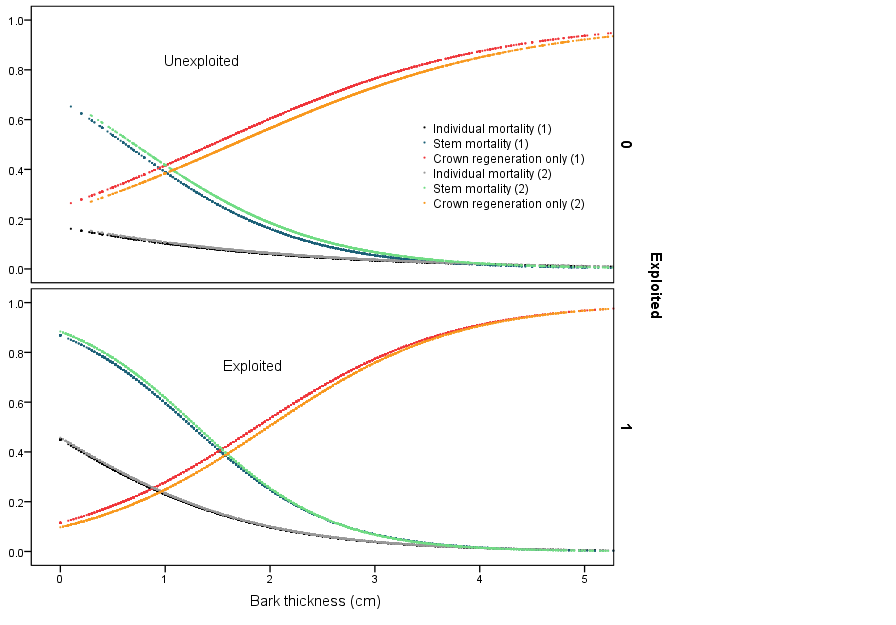


Fig. 1. Comparison of the post-fire *Q. suber* responses between the original (1) and the corrected (2) models presented in Table 2 (tree response probabilities are represented as a function of bark thickness and exploitation status; DBH is held constant at 21 cm, representing the average tree). For each response the differences between the two models are negligible.
